# Synergizing Chemical Structures and Bioassay Descriptions for Enhanced Molecular Property Prediction in Drug Discovery

**DOI:** 10.1021/acs.jcim.4c00765

**Published:** 2024-06-05

**Authors:** Maximilian G. Schuh, Davide Boldini, Stephan A. Sieber

**Affiliations:** TUM School of Natural Sciences, Department of Bioscience, Center for Functional Protein Assemblies (CPA), Technical University of Munich, 85748 Garching bei München, Germany

## Abstract

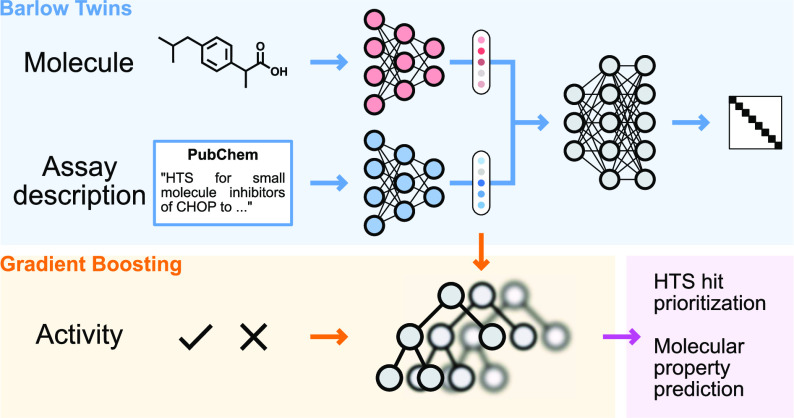

The precise prediction of molecular properties can greatly accelerate the development of new drugs. However, *in silico* molecular property prediction approaches have been limited so far to assays for which large amounts of data are available. In this study, we develop a new computational approach leveraging both the textual description of the assay of interest and the chemical structure of target compounds. By combining these two sources of information via self-supervised learning, our tool can provide accurate predictions for assays where no measurements are available. Remarkably, our approach achieves state-of-the-art performance on the FS-Mol benchmark for zero-shot prediction, outperforming a wide variety of deep learning approaches. Additionally, we demonstrate how our tool can be used for tailoring screening libraries for the assay of interest, showing promising performance in a retrospective case study on a high-throughput screening campaign. By accelerating the early identification of active molecules in drug discovery and development, this method has the potential to streamline the identification of novel therapeutics.

## Introduction

Quantitative structure–activity relationship (QSAR) modeling is a critical factor in accelerating the drug discovery and development process.^1−3^ Typical applications include ligand-based virtual screening, enabling the identification of bioactive compounds from large molecular libraries, and absorption, distribution, metabolism, excretion and toxicity (ADMET) property prediction.^4−6^

However, the data sets typically found in drug discovery are often not sufficiently large for developing accurate and reliable QSAR predictors.^7,8^ This is because many critical assays are time-consuming and expensive, especially for dose–response measurements, making the collection of a sufficient number of end points for QSAR modeling impractical.^9,10^ Furthermore, these data sets might lack chemical diversity, reducing the generalization ability of the resulting data-driven model, or might not report “negative” (e.g., inactive) compounds, limiting the amount of information available to train the QSAR model.^8,11^

To address the challenges arising from data scarcity in drug discovery, the joint modeling of chemical structures and bioassays is emerging as a promising solution.^3,12^ Instead of training to predict a specific property for a query molecule, these approaches receive as input both the chemical structure of the query molecule and the text description of the target assay. By training on a large collection of compound–assay pairs, the model can then be employed to predict bioactivity in new end points where no measurements are available, a process commonly referred to as zero-shot learning.^13^ This is analogous to proteochemometric modeling, where *in silico* predictors are trained on both molecular and protein embeddings, thus enabling bioactivity prediction of proteins not included in the original training set.^14^

In this work, we introduce a new zero-shot molecular property prediction approach named TwinBooster, which combines self-supervised learning (SSL), a large language model (LLM) and gradient boosting machine (GBM). TwinBooster is based on the Barlow Twins^15^ architecture, which enables the combination of textual and molecular information into useful features for QSAR modeling. The primary advantage of Barlow Twins over other SSL techniques lies in its novel objective function, which measures the cross-correlation matrix between the outputs of two identical networks processing different input representations. In our approach, these correspond to the text description of the assay of interest and the chemical information on the query compound. The aim is then to make this matrix as close as possible to the identity matrix, forcing different active compounds in a given assay to have similar representations as the text description of the experiment while minimizing feature redundancy.^15,16^

Our analysis shows that our method achieves state-of-the-art performance for zero-shot molecular property prediction, even outperforming alternative approaches that leveraged available measurements for a given end point. We then disentangle the impact each algorithmic component has on the performance of our approach, indicating that the performance improvement is spearheaded by the joint use of the Barlow Twins architecture and LLM modeling. Finally, we evaluate our proposed methodology on a retrospective case study tackling the issue of compound selection for high-throughput screening (HTS) campaigns. Our results show that despite only relying on the textual description of the assay, TwinBooster could correctly prioritize the hits of the screening campaign without compromising on chemical diversity.

## Results and Discussion

### Zero-Shot Prediction with TwinBooster

The key idea behind our method is to reframe the zero-shot prediction task as an assay–molecule matching operation, where the pipeline receives both data modalities as input and predicts the likelihood that the query molecule is active in the assay of interest.^13^ The main advantage of this approach is that it allows to use the wealth of bioassay data present in public repositories (e.g., PubChem)^17^ for pretraining, since the learning objective of the neural network is not specific to a given biological end point. Once the predictor is trained, it can be used to generate predictions on new molecules and assays by using it directly in inference mode. This is unlike alternative algorithms for few-shot prediction, which require fine-tuning on each new assay,^18−20^ making TwinBooster particularly attractive as an out-of-the-box solution for zero-shot prediction.

In practice, the TwinBooster framework is comprised of four building blocks.

• *A molecular featurization algorithm to convert chemical structures into numerical representations*. In this study, we employ extended-connectivity fingerprints (ECFPs),^21^ given their widespread popularity for QSAR modeling^22,23^ and their superior performance in other zero-shot learning studies.^12^
• *A scientific text encoder to convert bioassay descriptions and protocols into vectors*. For this application, we use the DeBERTA LLM architecture,^24,25^ fine-tuned on the large bioassay corpus from PubChem.^17^
• *A SSL architecture that pushes chemical and textual representations to be similar when a query compound is bioactive in a given assay*. Our approach employs the Barlow Twins architecture, a state-of-the-art neural network architecture for SSL tasks.^15^
• *A classification algorithm to predict whether a given compound is bioactive in the assay of interest, given a pair of molecular and textual features*. We opt for the LightGBM algorithm, given its computational efficiency and performance for QSAR modeling.^26−28^

**Figure 1:**
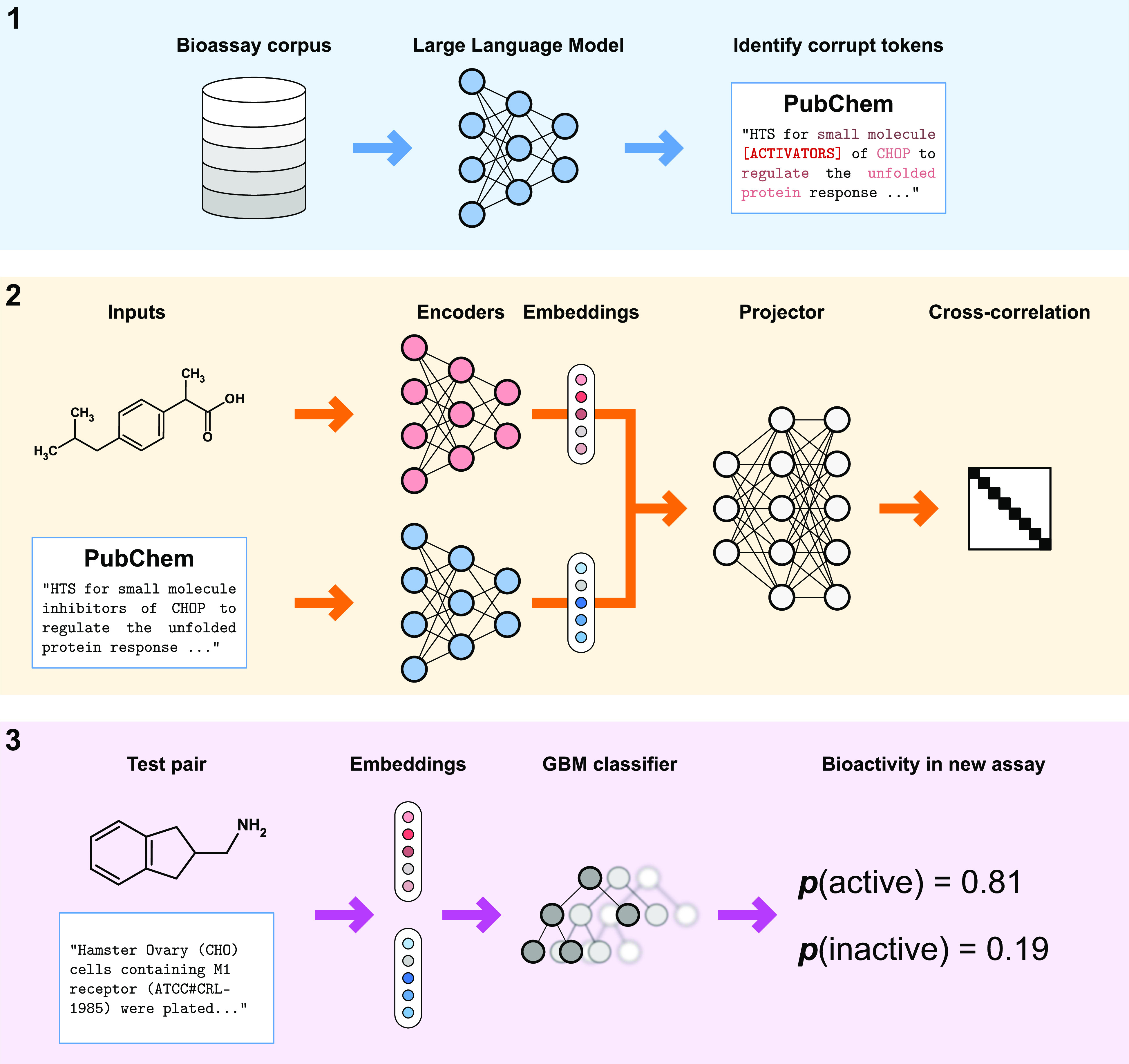
TwinBooster architecture. **(1)** The PubChem corpus is used to fine-tune a LLM.^17,24^**(2)** The Barlow Twins neural network provides a learned representation of molecules in the context of bioassays.^15^ It first independently encodes both molecular and textual information and then passes them through a unified projector. Finally, the model maximizes the diagonal terms of the cross-correlation matrix between projections, while minimizing the off-diagonal terms. **(3)** The learned embeddings are used to train GBM to predict whether a query molecule is active in the assay of interest.

The training procedure of TwinBooster can be summarized as a three-step process, shown in Figure 1. First, we fine-tune the LLM on bioassay protocols to improve the quality of the text embeddings in the drug discovery domain. Then, we train the Barlow Twins architecture to enforce that all bioactive compounds in the same bioassay have similar representations, improving their predictive power for zero-shot learning. Finally, we train a GBM classifier on a large collection of bioassay data to predict whether a given molecule–assay pair is a “match”, according to the features learned by the Barlow Twins architecture.

We describe in further detail the parameters used for each building block and the training procedure in the Supporting Information.

### Benchmark Structure

Given the algorithmic complexity of our approach, we perform several validation experiments to assess the performance of our proposed approach. First, we confirm that the fine-tuning procedure of the LLM provides better textual embeddings for the drug discovery domain.^29^ We then benchmark our proposed approach against FS-Mol,^20^ a comprehensive and well-established benchmark for evaluating molecular property prediction against several other algorithms in low-data settings.^12^ However, due to the modularity and complexity of our approach, it is difficult to determine which component is the main contributor of performance improvement. To address this, we conduct an ablation study to examine the impact of each building block on the performance of our method. Next, we investigate the applicability domain of our tool using conformal prediction, a popular algorithm for uncertainty quantification of machine learning algorithms.^30^ Finally, we benchmark TwinBooster in a use-case scenario for drug discovery, where the task is to correctly prioritize which compounds to screen first in a HTS campaign, given the experimental protocol of the assay of interest.

### Fine-Tuning the LLM Improves Bioassay Textual Data Embedding

While there are many LLMs available, none have been specifically developed for encoding bioassay protocols. Because of this, we fine-tune DeBERTa V3 on the corpus of assay descriptions from PubChem, referred below as “PubChemDeBERTa”.^17,24,25^ We detail the fine-tuning protocol in the Supporting Information. Adapting the LLM to the domain of interest should allow it to generate more informative text encodings for this domain, which in turn improves the predictive power of our proposed approach. To test this assumption, we compare the ability of our LLM to understand bioassay protocols with four established baselines: BERT,^31^ DeBERTa,^25^ DeBERTa V3^24^ and BioBERT.^32^ Perplexity, a common measure for evaluating the modeling performance of LLMs, is selected as the evaluation metric. In practice, this figure of merit quantifies how uncertain a LLM is at predicting a certain word, given its context in the sentence.^29,33^ The results are shown in Table 1.

**Table 1:** LLM Performance Evaluation by Looking at the Perplexity Towards the PubChem Corpus^a^

Model	Perplexity
PubChemDeBERTa	**1.52**
BioBERT^32^	4.47
DeBERTa V3^24^	10.7 × 10^6^
DeBERTa^25^	12.5 × 10^4^
BERT^31^	14.9

a The best value is highlighted in bold.

The analysis shows that our LLM outperforms all alternatives in modeling bioassay data from PubChem, achieving a perplexity value of 1.52. Both non fine-tuned DeBERTa models struggle to model assay descriptions, achieving perplexity values several orders of magnitude higher than all other alternatives (12.5 × 10^4^ and 10.7 × 10^6^ respectively).^24,25^ This is likely due to the fact that they rely on ELECTRA, a more advanced training procedure. Without fine-tuning, the additional complexity from ELECTRA pretraining could accentuate the issue of domain mismatch encountered by these models.^24^ However, if fine-tuning is performed, the ability of this neural network architecture to understand bioassay protocols greatly improves, as highlighted by the large performance improvement obtained by PubChemDeBERTa. Thanks to fine-tuning, our LLM also outperforms BioBERT,^32^ a well-established LLM for modeling biomedical textual data. This is likely due to the focus of our approach on exclusively the domain of interest, namely assay descriptions, as well as the superior performance of the DeBERTa architecture compared to BERT.^31^ Based on these considerations, our LLM is the best option for generating textual embeddings to be used for predicting activity in new bioassays according to the TwinBooster pipeline.

### TwinBooster Achieves Best Performance on FS-Mol

The FS-Mol repository is a popular benchmark for evaluating QSAR models in low-data scenarios, comprising of 122 assays and 27 363 unique compounds.^20^ It is typically used to evaluate QSAR approaches, either in the few-shot or zero-shot setting.^34,35^ The former measures the ability of a model to predict bioactivity in a target assay, given a handful of training samples (“support molecules”).^18,35^ The latter instead assesses the ability of an algorithm to predict bioactivity for end points never observed during training.^13,36,37^ Since the performance of QSAR models generally improves the more assay-specific data are used for training, zero-shot prediction is substantially more difficult than few-shot modeling.^13^ However, the ability to predict bioactivity without training data for that specific assay is more useful in practice, as it eliminates the need to perform any measurements beforehand.

We compare the performance of TwinBooster to a wide variety of deep learning (DL) architectures and machine learning (ML) approaches, including both zero-shot and few-shot settings. The few-shot baselines consist of prototypical network (PN),^38^ model-agnostic meta-learning graph neural network (GNN-MAML),^39^ multitask graph neural network (GNN-MT),^40,41^ random forest (RF),^42^ molecule attention transformer (MAT)^43^ and single-task graph neural network (GNN-ST).^20,40^ In terms of zero-shot performance, we compare our approach to CLAMP,^12^ a recently proposed state-of-the-art model for low-data molecular property prediction. This comparison is particularly meaningful because like our proposed approach, CLAMP also performs zero-shot prediction by combining modeling of the chemical and textual information for a given assay.^12^ Additionally, we include an additional baseline combining RF, ECFP and one-hot encoding of protein super family and assay type. However, since this approach cannot use all available training data due to missing annotations, we discuss it separately in the Supporting Information.

As indicated in the original FS-Mol publication, we measure performance in terms of precision recall area under curve (PR AUC) and Δ in precision recall area under curve (ΔPR AUC) to better compare model performance with random guessing.^20^ We describe the benchmarking protocol in more detail in the Supporting Information.

**Figure 2:**
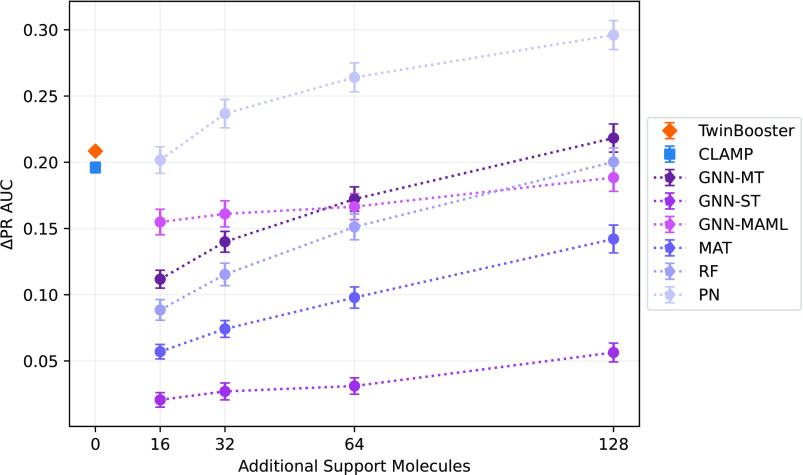
Zero- and few-shot FS-Mol benchmark performance of various ML/DL models.^12,20^ Standard deviations are shown between replicates.

Overall, TwinBooster shows excellent zero-shot performance, achieving a ΔPR AUC score of 20.84 ± 0.24% (Table 2). Crucially, it significantly outperforms all few-shot baselines up to 16 support molecules (Wilcoxon test,^46^ α = 0.05 with Benjamini-Hochberg^45^ multiple test correction), while remaining competitive with all other approaches (except PN) up to 128 training instances (Figure 2). This result is especially remarkable, since it highlights that TwinBooster is able to match or outperform alternative QSAR models without requiring any training data for a given assay. Additionally, TwinBooster also significantly outperforms CLAMP (ΔPR AUC of 19.37 ± 0.20%, Welch’s *t*-test,^44^ α = 0.05 with Benjamini-Hochberg^45^ multiple test correction).^12^ Compared to TwinBooster, CLAMP has two fundamental methodological differences. First, it relies on a different training objective. Second, it uses a different algorithm to generate bioassay protocol embeddings. As such, these results suggest that using the Barlow Twins architecture for SSL pretraining and modeling assay protocols with LLMs is beneficial.^15^ The latter point is consistent with the observation that both TwinBooster and CLAMP outperform the RF predictor based on protein family and assay one-hot encoding, highlighting the importance of correctly encoding the textual modality. To further investigate this research question, we analyze the impact of each module on the overall performance of TwinBooster through an ablation study.

**Table 2:** Comparing Different Zero- and Few-Shot Model Performances Across Different Metrics on FS-Mol^b^

Model	Mode	PR AUC (%)	ΔPR AUC (%)
**TwinBooster**	zero-shot	**68.56± 0.24**	**20.84±0.24**
**CLAMP**^a^^,^(12)	zero-shot	66.55 ± 0.20	19.37 ± 0.20
**PN**(20)	16-shot	67.72 ± 1.00	20.17 ± 1.00
**GNN-MAML**(20)	16-shot	63.04 ± 0.96	15.49 ± 0.96
**GNN-MT**(20)	16-shot	58.73 ± 0.67	11.17 ± 0.67
**RF**(20)	16-shot	56.40 ± 0.78	8.85 ± 0.78
**MAT**(20)	16-shot	53.25 ± 0.55	5.70 ± 0.55
**GNN-ST**(20)	16-shot	49.61 ± 0.55	2.06 ± 0.55

a Only means and standard deviations are given, not by task performance.

b In zero-shot mode, no “test” molecules are provided; in the case of the few-shot performance, 16 molecules of the “test” set are provided. 10 replicates are carried out for each task; the mean and standard deviation of means are reported. Results that are both the best and statistically significant (Wilcoxon test^44^ or Welch’s *t*-test^44^ in the case of CLAMP, α = 0.05 with the Benjamini-Hochberg^45^ multiple test correction) are highlighted in bold.

### Disentangling the Contributions of Each TwinBooster Component

To assess the impact of SSL pretraining and LLM embeddings on the performance of our approach, we evaluate how the performance of TwinBooster changes as more building blocks are removed from the pipeline. For this purpose, we repeat the evaluation procedure on the FS-Mol benchmark with the following modifications.

1. We remove Barlow Twins pretraining and we train the GBM classifier on ECFP and PubChemDeBERTa embeddings.^26−28^
2. From the prior ablation, we replace PubChemDeBERTa with latent semantic analysis (LSA), an alternative bioassay protocol encoder used in CLAMP.^12^

The first ablation shows that the performance significantly deteriorates without Barlow Twins embeddings (Wilcoxon test,^46^ α = 0.05 with Benjamini-Hochberg^45^ multiple test correction), dropping from 20.84 ± 0.24% to 20.41 ± 0.29% in terms of ΔPR AUC (Table 3). This result suggests that SSL pretraining is useful to better synergize the information provided by the textual and molecular modalities, leading to better zero-shot performance. Furthermore, given that TwinBooster also outperforms CLAMP, which relies on a different SSL architecture, this indicates that the Barlow Twins architecture is particularly effective at learning useful embeddings. This is likely because this DL architecture enforces that molecular and textual embeddings must be similar for bioactive compounds in a given assay, while simultaneously forcing the components of the representation to be orthogonal to each other. By optimizing both objectives simultaneously, the resulting encodings are similar for all bioactive compounds in a given assay while avoiding informational collapse, thus providing superior predictive performance.^15^

**Table 3:** Performance of Different Ablation Experiments on FS-Mol^a^

Ablations	PR AUC (%)	ΔPR AUC (%)
**TwinBooster**	**68.57±0.24**	**20.84± 0.24**
**ECFP + PubChemDeBERTa**	68.13 ± 0.29	20.41 ± 0.29
**ECFP + LSA**	67.51 ± 0.21	19.78 ± 0.21

a All results are tested pairwise using the Wilcoxon test with Benjamini-Hochberg multiple test correction.^44,47^ 10 replicates are carried out for each task; the mean and standard deviation of means are reported. Results that are both the best and statistically significant (Wilcoxon test,^44^ α = 0.05 with the Benjamini-Hochberg^45^ multiple test correction) are highlighted in bold.

The second ablation denotes another statistically significant performance decrease (Wilcoxon test,^46^ α = 0.05 with Benjamini-Hochberg^45^ multiple test correction), shifting from 20.41 ± 0.29% to 19.78 ± 0.21% in terms of ΔPR AUC (Table 3). This analysis highlights that LLM fine-tuning yields better embeddings for the bioassay protocol modality in terms of predictive power, consistent with the perplexity score obtained by our approach on the PubChem assay description corpus.

The change in performance is similar for both ablations, indicating that SSL pretraining and LLM fine-tuning contribute equally to the performance of TwinBooster. Thus, these results highlight that for QSAR modeling via multimodal learning, both encoding algorithms and SSL architecture should be optimized to maximize performance, indicating several avenues for further research.

Based on these considerations, these experiments confirm that the complex nature of our pipeline is justified, as each of the building blocks is critical to improving performance.

### Uncertainty Quantification Significantly Improves Performance

As TwinBooster can be used out-of-the-box to predict bioactivity in new assays, it is important to know when predictions are reliable. Therefore, we implement Mondrian conformal prediction into the pipeline, a popular approach for uncertainty quantification in molecular property prediction.^30^ For active and inactive molecules, predictions are made on a calibration set, then confidence scores are assigned and the corresponding thresholds can be determined. If a prediction falls within the class-specific threshold, it is considered to be confident. To investigate whether conformal prediction captures the prediction uncertainty of TwinBooster, we compare the performance of our tool on FS-Mol when focusing on confident predictions. Across all metrics, performance could be significantly improved, e.g. with a relative ΔPR AUC increase of ∼10% (shown in Table 4). The proportion of predictions that are confident out of all predictions across all bioassays is 65%, promoting the usability of our pipelines. This shows that confident predictions are more accurate, i.e. conformal prediction correctly identifies the applicability domain while retaining two-thirds of the predictions.

**Table 4:** Comparing Zero-Shot Performances with or without Conformal Prediction on FS-Mol^a^

Conformal Prediction	PR AUC (%)	ΔPR AUC (%)
with	**71.04±0.31**	**22.81±0.30**
without	68.56 ± 0.24	20.84 ± 0.24

a The confidence level is set to ϵ = 0.80. 10 replicates are carried out for each task; the mean and standard deviation of means are reported. Results that are both the best and statistically significant (Wilcoxon test,^46^ α = 0.05) are highlighted in bold.

### Exploring the Challenges of Interfering Compounds in HTS

HTS data sets are notoriously noisy due to the widespread presence of frequent hitters, assay artifacts and promiscuous compounds such as pan-kinase inhibitors. Frequent hitters are compounds that appear to be active in multiple assays, often due to nonspecific interactions rather than true biological relevance.^48,49^ Assay artifacts, such as autofluorescence, redox cycling, colloidal aggregation, etc., produce a positive readout without eliciting the desired biological response.^50,51^ Finally, undesirable polypharmacology can also complicate the hit selection process in HTS data, such as in the case of pan-kinase inhibitors.^52−54^ In the following section we investigate whether the predictions of TwinBooster are affected by these artifacts.

To assess whether TwinBooster predicts frequent hitters, all molecules are processed using HitDexter 3, a well-established frequent hitter identification software.^55^ We then perform a correlation analysis between the predictions from TwinBooster and the frequent hitter probability according to HitDexter 3. The mean Pearson correlation coefficient^56^ is ρ = 0.0359 ± 0.0524, indicating no correlation, thus showing that the predictive performance of our approach is not due to a bias toward frequent hitter chemical moieties.

Next, we analyze whether the predictions of TwinBooster are correlated to the presence of GSK and REOS structural alerts, two commonly used rule-based approaches to detect artifacts in HTS data.^57^ A ROC AUC ≃ 0.5179, while a ROC AUC = 0.5 denotes randomness, also indicates no correlation between TwinBooster predictions and potential assay artifacts (Figure S2).

**Figure 3:**
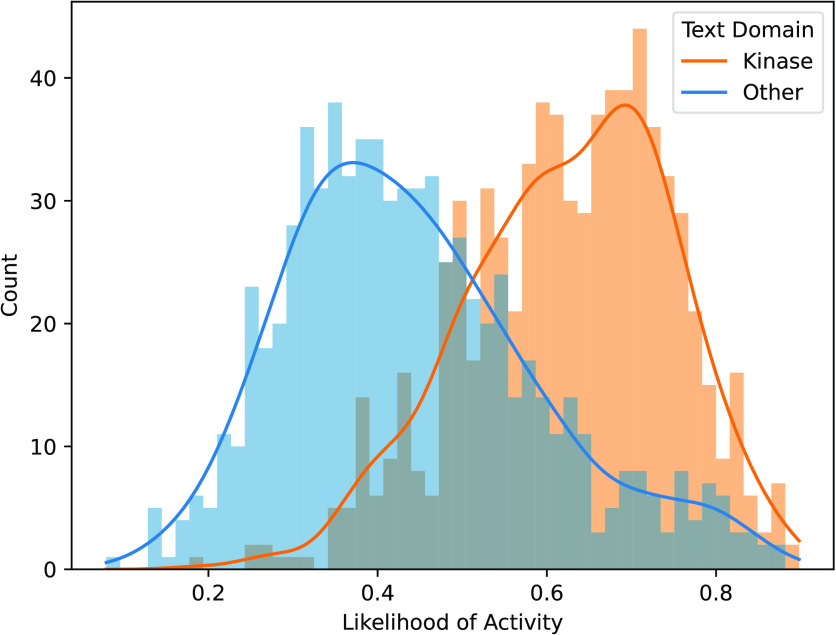
Comparison of the likelihood of activity between text domains. Ten assay texts are randomly sampled from the FS-Mol test set per text domain class.

Finally, we investigate whether TwinBooster is biased by the bioassay text domain used for pretraining. Since the largest annotated protein class of the FS-Mol training data consists of kinases, TwinBooster could theoretically achieve good predictive performance on this benchmark by focusing on identifying pan-kinase inhibitors, regardless of the provided bioassay description. To test this hypothesis, we identified potential pan-kinase inhibitors in our training data, as done by Lin et al.,^58^ yielding 73 candidate compounds. If TwinBooster ignores the provided text modality, these molecules should be always predicted active regardless of text modality. Therefore, we investigate whether predictions change when replacing the original text input with other bioassay descriptions. Figure 3 shows that the predictions of TwinBooster reflect the text domain change, as the pan-kinase inhibitors are classified as inactive when paired with nonkinase assays, while they are predicted to be broadly active in kinase-related assays.

As a further ablation, we built a model trained only on the kinase information contained in the FS-Mol training set. We analyze the performance on the test tasks, which are nonkinase assays, and are able to show that TwinBooster is not only driven by the kinase information, but also shows drastically improved performance on the diverse protein family tasks compared to the kinase-only model (refer to Figure S3).

### Case Study: Using Zero-Shot Learning to Tailor a HTS Screening Library

An appealing application of TwinBooster is the tailoring of a HTS library according to the description and experimental protocol of the target assay. To investigate this capability, we perform a retrospective analysis of a published HTS campaign.

We chose the HTS campaign from Flaherty et al., aimed at identifying compounds that selectively activate the C/EBP homologous protein (CHOP) pathway in the context of endoplasmic reticulum (ER) stress. This pathway becomes particularly relevant in pathological conditions such as cancer, where ER stress and unfolded protein response (UPR) dysregulation are often observed. We focused on this data set for the following reasons.

• Both primary and confirmatory screening data are available, so that the analysis is not biased by HTS false positives.^60−62^
• All associated data are publicly available on PubChem.
• This assay is not included in the training set of our tool or in FS-Mol. This ensures that this case study is an unbiased evaluation of the prospective performance of TwinBooster.

This screening campaign led to the discovery of a class of sulfonamidebenzamide compounds that effectively activate the CHOP pathway. Subsequent investigations, including structure–activity relationship studies, allowed these compounds to be optimized for improved potency and selectivity.^59^

**Figure 4:**
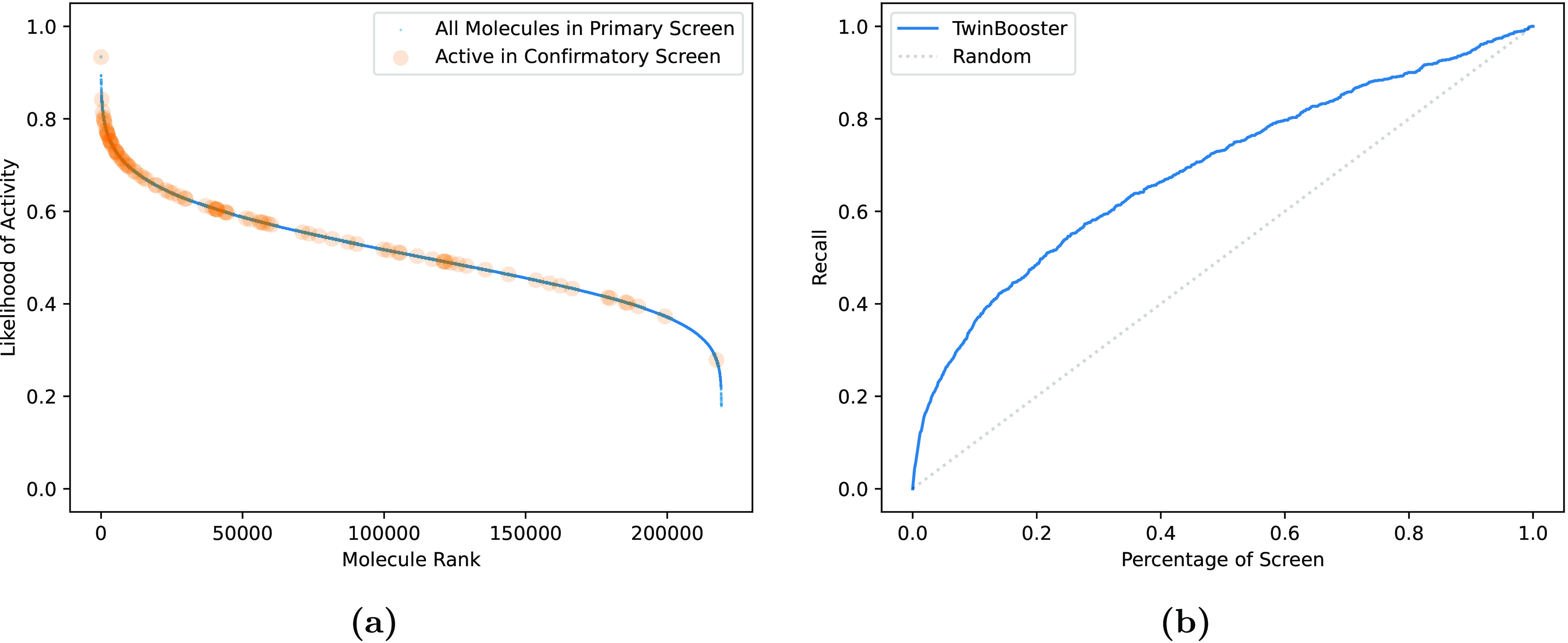
Zero-shot predictions on the primary screen of the case study. (a) Ranked molecules based on zero-shot prediction: highlighting earlier discovery of confirmatory screening hits. (b) Recall curve: retrieved active compounds as a percentage of all active compounds based on the percentage of the primary screen provided.

The aim of this case study is therefore to assess how well TwinBooster could have identified the validated HTS hits from this campaign, relying solely on the assay protocol description, before performing any practical experiment. First, we measured the Boltzmann-enhanced discrimination of receiver operating characteristic (BEDROC) score (α = 20), an established “early detection” metric,^63^ to evaluate the ranking performance of our approach on this screening campaign. TwinBooster achieves a BEDROC of 0.1768, corresponding to an improvement over random sampling resulting in a BEDROC of 0.1069.^63^ The BEDROC score can be further increased by focusing on confident predictions, achieving a score of 0.2254. This indicates that our tool correctly prioritizes the majority of HTS hits. The performance of TwinBooster can also be intuitively visualized in Figure 4a, which shows that most HTS hits are predicted to be highly likely to be active. We further quantified the performance of our approach by plotting the recall curve obtained by TwinBooster (Figure 4b). In practice, this analysis describes what fraction of the total HTS hits are retrieved as more compounds are selected for testing according to the predictions of our method. For example, when evaluating the top 20% of TwinBooster’s predictions, it is possible to identify 49% of all active compounds, further demonstrating the good prioritization performance of the tool.

**Figure 5:**
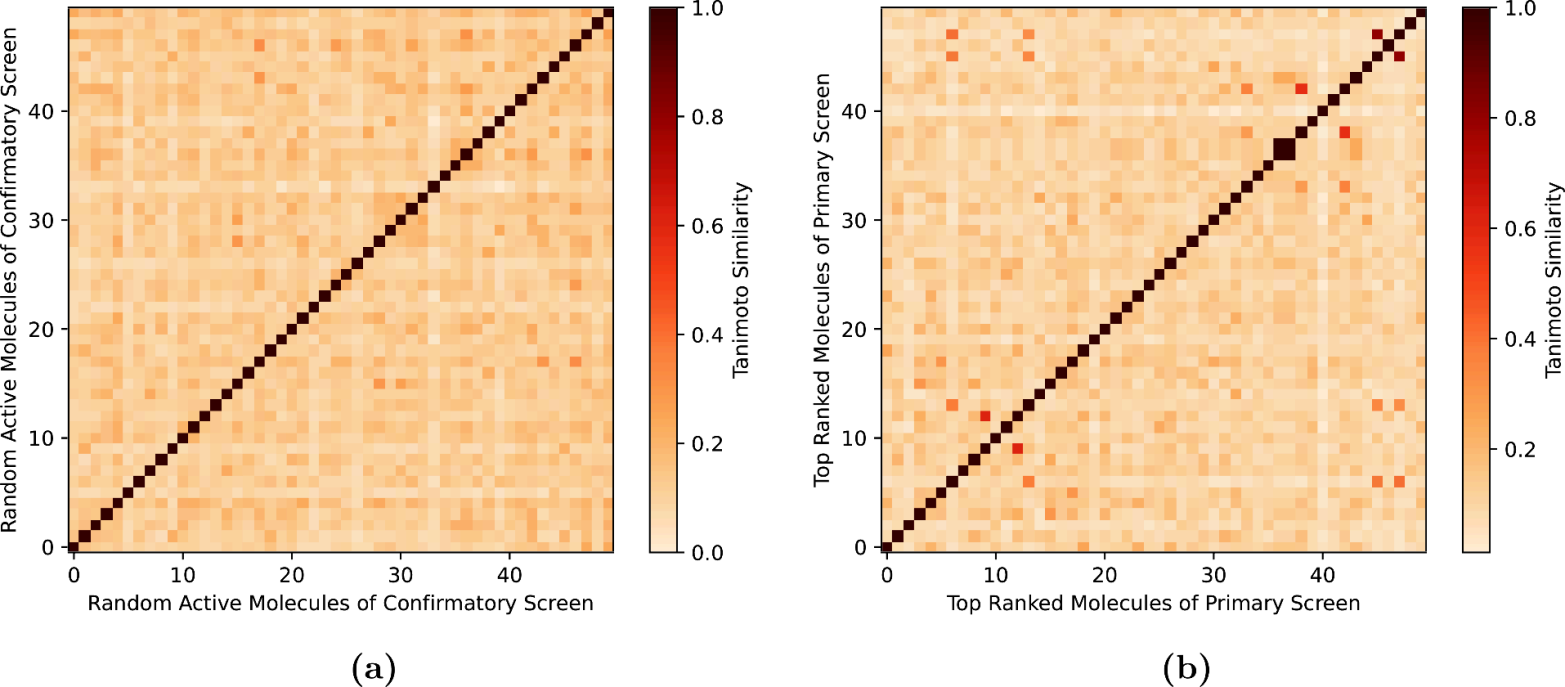
Tanimoto similarity compound selection to highlight structural similarities and differences. (a) 50 active compounds are sampled from the confirmatory screen. (b) The top 50 compounds are selected based on the TwinBooster ranking of the primary screen.

Finally, to investigate whether the performance is due to a bias toward specific chemical scaffolds, we evaluated the molecular diversity of the hits identified by our approach. For this purpose, we compared the mean Tanimoto similarity (using ECFPs as molecular features) of the top 50 ranked molecules according to TwinBooster versus the one measured for 50 random hits from the HTS campaign (Figure 5). The analysis shows that the TwinBooster hits are significantly more diverse (Mann–Whitney *U* test,^44^ α = 0.05) than randomly selected compounds, indicating that TwinBooster provides a chemically unbiased compound prioritization strategy.

The low mean Tanimoto similarity of TwinBooster hits (0.11 ± 0.06) is consistent with the number of unique Murcko scaffolds identified at different points in the ranking (Figure S5). For example, the top 20% of compounds according to TwinBooster contain 34% of the unique Murcko scaffolds in the screening campaign, further demonstrating an enrichment of structural diversity.^57^ In conclusion, this case study demonstrates that TwinBooster can successfully prioritize the most likely active compounds in a screening campaign given the protocol of the assay of interest. As such, this tool can be used to identify privileged subsets of HTS libraries according to the assay of interest, thus accelerating the hit identification process in drug discovery.

## Conclusions and Outlook

By integrating a fine-tuned LLM, the Barlow Twins SSL architecture and GBM, our TwinBooster framework represents a novel approach to molecular property prediction for scenarios where data is scarce.

Our approach achieved excellent performance on the FS-Mol benchmark, outperforming several few-shot QSAR models without requiring any training data for the assay of interest. Furthermore, our algorithm outperformed CLAMP, an alternative zero-shot learning approach based on joining textual and molecular input modalities for QSAR modeling. This result, combined with the ablation study we conducted, demonstrates the impact of tailored SSL strategies and encoder architectures on zero-shot learning and suggests further research directions.

Additionally, when applied to a retrospective HTS case study, our model could effectively identify active compounds, showing the potential of tailoring screening libraries by relying exclusively on the assay protocol.

Finally, we have made this tool accessible through an easy-to-use Python notebook that provides a straightforward way for users to input bioassay data and molecules for prediction and uncertainty estimation.

Given its excellent performance and usability, we are confident that TwinBooster can help accelerate HTS campaigns by allowing researchers to focus on the most relevant compounds in the screening library, thus reducing the time and cost associated with hit identification in drug discovery.

## Methods

### Data Set

The FS-Mol data set presented by Stanley et al. proposes a new binary classification data set and benchmarking procedure in drug discovery. It is focused toward (zero-) and few-shot learning, to analyze small data sets, which are common in drug discovery due to high data generation costs and ethical considerations.^20^ FS-Mol contains multiple few-shot ML baselines, additionally to literature where zero-shot learning is introduced.^12,35^ It provides predefined per-task training, validation as well as testing splits, sourced from ChEMBL.^64^ The test split is withheld from training and used only for performance evaluation. FS-Mol provides simplified molecular-input line-entry system (SMILES) and the true label per molecule. All molecules are converted to ECFPs 1024 bits and a radius of 2, using the Python^65^ Rdkit^57^ implementation. Each task is only contained in a single split.^20^

#### Bioassay-Based LLM Text Embeddings

We acquire titles, descriptions and protocols as additional representation for each molecule and assay. This assay text information is retrieved from PubChem mapped to the ChEMBL-based FS-Mol benchmark.^17,64^ For TwinBooster text information is processed using the fine-tuned PubChemDeBERTa LLM.

#### Performance Evaluation

The performance of each FS-Mol task is measured using PR AUC and ΔPR AUC, this is performed in 10 replicates. The reported performance is calculated by averaging across replicates and then across tasks. Statistical testing is performed by using either Wilcoxon signed-rank test, Welch *t*-test or Mann–Whitney *U* test, in combination with Benjamini-Hochberg multiple test correction if necessary (further described in SI “Statistical testing”).

### Models

#### Language Models

##### Fine-Tuning Large Language Models

In this study, we used Microsoft’s developed DeBERTa V3 base^24^ as the underlying architecture for our LLM and fine-tuned it on a comprehensive bioassay corpus obtained from PubChem.^17,24,25^ We chose DeBERTa V3, a pretrained LLM, for this research due to its superior performance compared to the original DeBERTa or BERT model, respectively.^24^ The description is shuffled (“.” as delimiter) with 5 augmentations as the training corpus and then fine-tuned using the Python^65^ Transformers^66^ library. Hyperparameters are optimized with Optuna^67^ for 20 trials (ref table S1, S2).

##### LLM Baselines

For performance comparisons on the PubChem corpus,^17^ this study employs several existing LLMs including BERT base uncased,^31^ DeBERTa base,^25^ DeBERTa V3 base^24^ and BioBERT V1.2 base cased.^32^

##### Latent Semantic Analysis

The latent semantic analysis (LSA) model^68^ is pretrained on the PubChem data set, utilizing the TfidfVectorizer and TruncatedSVD Python packages as outlined by Seidl et al.^12,69^

##### Performance Evaluation

For evaluation, we use perplexity, a metric that indicates the performance of the LLM (lower values represent superior performance).^29,33^ The premise is that a model’s ability to predict subsequent words in new sentences indicates a solid understanding of the drug discovery language. We perform the LLM evaluation on a test set comprising 20% of the corpus, with a token corruption rate of 15%, using the PubChem corpus.^17^

### TwinBooster

#### Multilayer Perceptron

The Barlow Twins model uses multilayer perceptrons (MLPs) in both encoder and projector designs, modifying the original architecture to include a molecule and a text encoder, and employing ECFPs and PubChemDeBERTa embeddings.^15^ This model is trained on both negative and positive samples to enhance generalization and uses a shared projector for both representations. It is developed using PyTorch.^70^

Training involves the Barlow Twins loss^15^ with the AdamW optimizer.^71^ It includes manual hyperparameter adjustments as detailed (in table S3) and continues for 25 epochs or until early stopping is triggered.

#### Gradient Boosting Machine

The gradient boosting machine (GBM) uses LightGBM from the Barlow Twins model’s information bottleneck embeddings for training.^72^ Zero-shot predictions use ECFPs and textual data about target molecules.^26^ To improve performance, the SMAC3 library^73^ optimizes hyperparameters over 200 trials using 80% of the training data from the FS-Mol data set (see table S4). Evaluations of PR AUC and ROC AUC are performed on both the validation and the remaining 20% of the training data. SMAC3 facilitates this with its multifidelity implementation using the n_estimators parameter of LightGBM.^26,73^

## Data Availability

The system used for computational work has an AMD Ryzen Threadripper PRO 5995WX CPU with 64/128 cores/threads with 1024 GB RAM. Additionally, the server is equipped with a NVIDIA RTX 4090 GPU with 24 GB VRAM. The fine-tuned model on the PubChem corpus, PubChemDeBERTa, is available on HuggingFace https://huggingface.co/mschuh/PubChemDeBERTa-augmented. As a Python package, it can be installed using $ pip install twinbooster. The code is available on GitHub https://github.com/maxischuh/TwinBooster, where you can also find the model data.

## Supporting Information Available

Deeper technical explanation of TwinBooster, the Additional Methods section details how each approach was implemented, LLM, Barlow Twins and GBM construction, respective hyperparameter tuning, conformal prediction, *p*-values of statistical testing, and chemical space diversity analysis (PDF)
