# Supplementary material for: Synergizing Chemical Structures and Bioassay Descriptions for Enhanced Molecular Property Prediction in Drug Discovery
Source: J Chem Inf Model. 2024 Jun 5;64(12):4640–50. doi: 10.1021/acs.jcim.4c00765 (PMC11200265; doi:10.1021/acs.jcim.4c00765)
Supplement: Supplementary file 1 — ci4c00765_si_001.pdf [file ci4c00765_si_001.pdf]

Supporting Information: Synergising Chemical Structures and Bioassay Descriptions for Enhanced Molecular Property Prediction in Drug Discovery

Maximilian G. Schuh, Davide Boldini,* and Stephan A. Sieber*

Technical University of Munich, TUM School of Natural Sciences, Department of Bioscience, Center for Functional Protein Assemblies (CPA), 85748 Garching bei München, Germany

E-mail: davide.boldini@tum.de; stephan.sieber@tum.de

Additional Methods

Dataset

FS-Mol The FS-Mol dataset[S1] proposes a new approach to drug discovery using few-shot learning, to analyse small datasets, which are common in drug discovery due to high data generation costs and ethical considerations. The classification dataset and benchmarking procedure are designed to simulate the challenges of machine learning in drug discovery, where typically only a few hundred compounds can be tested. FS-Mol evaluates single-task, multi-task and meta-learning approaches and contains machine learning (ML) baselines. It provides training, validation as well as testing data, which are sourced from ChEMBL.[S2] In the context of few-shot learning a set from 16 up to 256 support molecules, alongside binary activity labels are provided.[S1]

It should be noted that 67 % of the FS-Mol data is not annotated with target protein information. The distribution of protein families present in FS-Mol is shown in fig. S1.

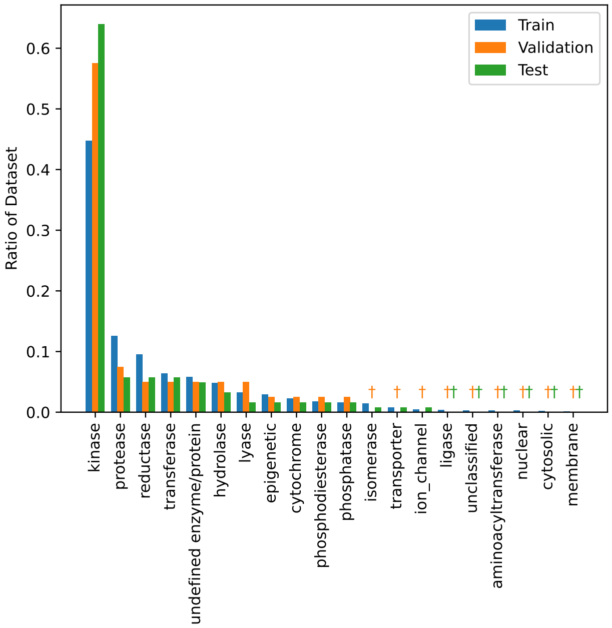

Figure S1: Normalised ratios of annotated tasks contained in the splits of FS-Mol according to their protein super family. The dagger denotes a missing protein family.

Molecular representation

Bioassay-based large language model (LLM) text embeddings The pipeline in this study requires titles, descriptions and protocols as additional representation for each molecule and assay. Therefore, this text information is extracted from PubChem.[S3] Using both Application programming interfaces (APIs) from PubChem and ChEMBL a mapping of bioassay identifier (AID) to ChEMBL IDs is performed.[S2,S3] This is done to retrieve the information rich text information of PubChem in combination with the ChEMBL-based FS-Mol benchmark. Only the 122 overlapping tasks of the test set could be mapped using the API of PubChem and ChEMBL, where the FS-Mol data originate.

The API matched AIDs are 521, 689, 881, 883, 899, 1215, 1394, 1540, 1708, 1750, 2161, 2230, 2364, 2572, 31668, 48288, 52163, 218702, 310904, 404304, 449749, 456868, 463120, 482894, 485349, 485367, 488785, 488789, 488835, 488921, 493182, 493248, 504729, 507074,

507077, 588344, 588345, 588811, 602234, 602235, 602374, 602386, 652135, 720021, 720033, 720044, 720046, 720076, 720081, 720107, 720113, 720115, 720127, 720130, 720132, 720134, 720136, 720137, 720146, 720157, 720162, 720163, 720175, 720180, 720185, 720189, 720191, 720200, 720202, 720207, 720233, 720237, 720246, 720248, 720251, 720261, 720262, 720267, 720276, 720278, 720281, 720285, 720289, 720290, 720295, 720298, 720310, 720312, 720319, 720323, 720329, 720331, 720339, 720354, 720357, 720359, 720361, 720370, 720373, 720384, 720392, 720395, 720421, 720422, 720427, 720439, 720442, 720445, 720446, 720450, 720453, 720463, 720473, 720478, 720481, 720482, 1053173, 1207589, 1207591, 1207592, 1438147 and 1501337.

Finally, the text is converted into a vector (of shape 768) using our fine-tuned LLM PubChemDeBERTa-augmented.

Extended-connectivity fingerprints All molecules are handled in simplified molecular-input line-entry system (SMILES) strings then converted to ECFPs 1024 bits and a radius of 2, using the Python[S4] Rdkit[S5] implementation.

Frequent hitter and assay artefact analysis

We apply GSK and REOS structural alerts using Rdkit.[S5] The more matches a compound has with these filters, the higher its chance of being a false positive. We categorise in two classes, compounds with no alerts and compounds with one or more alerts.

We use the HitDexter 3 webserver (`https://nerdd.univie.ac.at/hitdexter3/`)for batch processing to predict frequent hitters.[S6] We analyse the correlation of the pairs of TwinBooster predictions in combination with the likelihood of being a highly promiscuous compound. The data is extracted from these columns `HPROM-NPROM target-based assay data set model`, `HPROM-NPROM cell-based assay data set model` or `HPROM-NPROM extended cell-based assay data set model` of the `.csv` output from the webserver.[S6]

To investigate the performance of a kinase-only model a random forest (RF) model

is trained on the kinase subset of the FS-Mol training set (based on the protein super family).[S1,S7]

Case study

To highlight the zero-shot capabilities of TwinBooster a case study of biological high-throughput screening (HTS) is conducted. Therefore, the primary screen (AID 2732*) is analysed by TwinBooster to predict the desired properties. Then it is analysed against the confirmatory screen (AID 504437†). The columns `PUBCHEM_EXT_DATASOURCE_SMILES`, `PUBCHEM_ACTIVITY_OUTCOME` as well as the text information are used for the TwinBooster prediction pipeline.[S3]

Performance evaluation Recall measures the proportion of relevant instances that are retrieved, this refers to the active compounds in this case study.[S8] It can be expressed at the ratio of the found active molecules (true positives (TPs)) and all active molecules (TPs and false negatives (FNs)).

Similarity estimation The Tanimoto similarity of compounds is calculated using the corresponding Rdkit function.[S5] To highlight structural similarities and differences, 50 active compounds are sampled from the confirmatory screen and compared to the top 50 compounds are selected based on the TwinBooster ranking of the primary screen.

Models

Language models

Fine-tuning large language models In this study, we used Microsoft's developed DeBERTa V3[S9] as the underlying architecture for our LLM and fine-tuned it on a comprehensive bioassay corpus obtained from PubChem.[S3,S9,S10] We chose DeBERTaV3, a pretrained LLM,

*https://pubchem.ncbi.nlm.nih.gov/bioassay/2732
†https://pubchem.ncbi.nlm.nih.gov/bioassay/504437

for this research due to its superior performance compared to the original DeBERTa or BERT model, respectively.[S9] DeBERTa V3 uses the replaced token detection pretraining task, which is more sample-efficient than the traditional masked LLM approach. This innovation enhances both training efficiency and model quality by removing the "tug-of-war" dynamics present in the vanilla embedding sharing method used in ELECTRA.[S9,S10] The fine-tuning process aimed to enhance the model's performance in the context of biomolecular properties.

The DeBERTa V3 base model[S9] is fine-tuned on the PubChem corpus using ∼14 GB video random-access memory (RAM) for ∼15 h. In the augmented version, the description is shuffled ("." as delimiter) and 5 augmentations are used as the training corpus. Therefore, the Python[S4] Transformers[S11] library is used. The Optuna[S12] hyperparameter optimisation library is used to find the best combination of hyperparameters for the LLM (ref. table S1). After 20 optimisation procedure trials the best hyperparameters shown in table S2 were found.

Table S1: LLM hyperparameter optimisation space.

**Hyperparameter**	**Range**
learning_rate	$\{1.5 \times 10^{-5}, 2 \times 10^{-5}, 2.5 \times 10^{-5}, 3 \times 10^{-5}\}$
batch_size	{16, 32}
max_length	{64, 128}
num_train_epochs	1.0

Table S2: Best LLM fine-tuning hyperparameters.

**Hyperparameter**	**Value**
ampere	True
num_train_epochs	3.0
learning_rate	3×10^{-5}
weight_decay	0.01
batch_size	32
max_length	128
adam_beta1	0.9
adam_beta2	0.999
adam_epsilon	1×10^{-6}
warmup_steps	500

LLM baselines To compare with existing LLMs BERT base uncased,[S13] DeBERTa base,[S10] DeBERTa V3 base[S9] and BioBERT V1.2 base cased[S14] are used for performance comparisons on the PubChem corpus.[S3]

One-hot encoding To assess a rule-based baseline to ML or deep learning (DL) encoding, we use one-hot encoding for the assay type and protein super family information. To each ECFP of the contained molecules we add 42 one-hot encoded features, that are task specific. This method only can be applied where FS-Mol tasks are annotated which is only the case for 33 % of all tasks. Information is taken from the `target_info.csv` file found in the FS-Mol repository.

Latent semantic analysis The latent semantic analysis (LSA) model[S15] is pretrained on the PubChem corpus. This process is carried out in a manner similar to the approach used in Seidl *et al.*, using the Python packages `TfidfVectorizer` and `TruncatedSVD`.[S7,S16] The resulting text embeddings for each bioassay have a dimensionality of 355.

Performance evaluation For the evaluation, the perplexity (PPL) is used as an evaluation metric for fine-tuning the LLM (PPL $\in [0, \infty[$, lower values indicate better performance).[S17,S18] The underlying hypothesis is that the ability of a model to predict subsequent words in unseen sentences with a high probability indicates a comprehensive understanding of the grammatical structures of the language. The LLM evaluation is performed on the test set (20 % split) of the corpus with a token corruption rate of 15 %. The training and evaluation procedures are conducted using the PubChem corpus.[S3]

Perplexity is mathematically defined as the exponential of the cross-entropy loss between the predicted word probabilities and the actual distribution in the test set. Given this definition, the perplexity of the model can be expressed as: $\text{PPL} = \exp(H(p, q)) = \exp(\texttt{eval\_loss})$ where $H(p, q)$ denotes the cross-entropy loss.

TwinBooster

Multilayer perceptron Barlow Twins use multilayer perceptrons (MLPs) for both the encoders and the projector design.[S19] The network architecture is altered from the original by having two encoders a molecule and a text encoder. The Barlow Twins model trains on negative as well as positive examples, which should help generalisation. Finally, the projector is shared for both representations.

Both encoders as well as the projector have the following structure

$$\boldsymbol{l_{i+1}} = \mathrm{Linear}\left(\phi\left(\mathrm{BatchNorm}\left(\mathrm{Linear}\left(\boldsymbol{W}\boldsymbol{l_i} + \boldsymbol{b}\right)\right)\right)^n\right),$$

where $\boldsymbol{l_i}$ is the input layer and $\boldsymbol{l_{i+1}}$ is its output, with a flexible number of layers n and adjustable dimensionality of input and output. Furthermore, variables $\boldsymbol{W}$, $\boldsymbol{b}$ represent learnable weights and biases. A linear layer is followed by batch normalisation,[S20] an activation function ϕ,[S21,S22] and the last linear layer. The network is constructed using PyTorch.[S23]

For training the network is using Barlow Twins loss[S19] and the AdamW optimiser.[S24] Manual hyperparameter tuning is performed on a range and set of parameters listed in table S3. The model is trained for 25 epochs or until early stopping is engaged if a validation set is provided.

Furthermore, the model is trained using ECFPs and LLM embeddings. For inactive molecules, the embeddings are sign changed.

Gradient boosting machine The gradient boosting machine (GBM) package LightGBM is used for for training based on the informational bottleneck embeddings provided by the Barlow Twins model.[S25] Performing zero-shot predictions is done by feeding the Barlow Twins model with ECFPs and text information of the desired molecules.[S26] To achieve optimal performance, the SMAC3[S27] multi-fidelity hyperparameter optimisation library is applied to find the optimal combination of hyperparameters (ref. table S4), using 80 % of the "train"

Table S3: Barlow Twins Hyperparameters. The set of parameters is listed and the best are highlighted in bold.

**Hyperparameter**	**Set**
enc_n_neurons	{512, 1024, 2048, **4096**, 8192}
enc_n_layers	{2, 3, **4**}
proj_n_neurons	{512, 1024, **2048**, 4096, 8192}
proj_n_layers	{**2**, 3, 4}
embedding_dim	{512, **1024**, 2048, 4096}
act_function	{ReLU,[S21] **Swish**[S22]}
batch_size	{**1024**, 2048}
learning_rate	$\{5 \times 10^{-3}, \mathbf{1 \times 10^{-4}}\}$
weight_decay	$\{1 \times 10^{-3}, \mathbf{5 \times 10^{-3}}\}$

data of the FS-Mol dataset for training. Optimisation is set to 200 trials. The evaluation is performed by assessing the precision recall area under curve (PR AUC) and receiver operating characteristic area under curve (ROC AUC) on the "valid" and the remaining 20 % of the "train" data of the FS-Mol benchmark. SMAC3's multi-fidelity implementation is used with the budget parameter being represented by the n_estimators parameter of LightGBM.[S26,S27]

Table S4: GBM SMAC3 hyperparameter optimisation space.

**Hyperparameter**	**Range**
budget (n_estimators)	$[200, 2000]$
num_leaves	$[62, 256]$ (step size 64)
learning_rate	$[1 \times 10^{-8}, 1.0]$ (log scale)
min_child_samples	$[5, 100]$
subsample	$[0.4, 1.0]$
subsample_freq	$[0, 7]$
reg_lambda	$[1 \times 10^{-8}, 10.0]$

Finally, the LightGBM is trained using the full "train" data of the FS-Mol dataset and the best hyperparameters listed in table S5.

Performance evaluation When comparing models, we are using intersecting tasks of the "test" data, to ensure a scientific comparison. Metric selection is based on the FS-Mol benchmark.[S1] In addition, ROC AUC is commonly used for classifier evaluation in the

Table S5: Best GBM hyperparameters after optimisation.

Hyperparameter	Value
budget (n_estimators)	2000
num_leaves	256
learning_rate	0.0711
min_child_samples	60
subsample	0.941
subsample_freq	1
reg_lambda	3.78

presence of class imbalance, but it can be less reliable for rare classes due to small sample sizes.[S8,S28]

PR AUC is recommended for highly skewed classes, as it provides a more realistic view of classifier performance than ROC AUC.[S8,S29–S31] Moreover, both metrics can be calculated based on the probability of the prediction rather than the prediction itself, where a classification threshold problem can arise.[S8]

In the context of zero and few-shot learning, a different form of PR AUC, known as

$$\Delta \text{PR AUC}\,(t_i) = \text{PR AUC}\,(t_i) - \frac{\sum t_i}{|t_i|},$$

is used, where t_i denotes a particular task or bioassay within the total set of i tasks. The expression $\sum t_i$ represents the sum of all activity endpoints for a given task, indicating the number of active molecules. In addition, $|t_i|$ corresponds to the size of the task. This metric shows sensitivity to the balance between classes, allowing for straightforward comparisons with a baseline benchmark. This is due to the performance of a random classifier reflecting the percentage of positive endpoints.[S1] This metric is also used in the FS-Mol benchmark.[S1]

Conformal prediction In our study, we apply the conformal prediction method using the LightGBM classifier.[S26,S32] This technique involves a two-step process: calibration with cross-validation on training data (5 fold), because no calibration set is provided, and prediction on

test data.[S33] Then GBM predictions are analysed while the confidence level is set to $\epsilon = 0.80$. This method is valuable in providing both predictive outputs and insights into the certainty of each prediction.[S32]

Statistical testing

Whenever possible, the two-sided Wilcoxon signed-rank test is used in this study due to the non-parametric nature of the populations.[S34] When comparing means and standard deviations, the Welch t-test is used.[S34] The Mann-Whitney U test is performed when assessing whether Tanimoto similarities of two independent samples come from the same population.[S34] A correction for multiple testing is applied if necessary. The Benjamini-Hochberg multiple test correction is used for performance comparisons.[S35]

The seed is set to 42 where possible, or to the number of the performed replicate (seeds := $[0..n_r[$). 10 replicates per model are performed.

Additional Results

Fine-tuned LLM

The augmented version is referred to in the main text as "PubChemDeBERTa". The non-augmented version could have a perplexity of 2.32, while with the augmentation the perplexity could be reduced to 1.52.

Zero-shot benchmark

The zero-shot performances of 10 replicates are averaged and compared with baselines from FS-Mol.[S1] The p-values are shown in table S7.

[‡]Only means and standard deviations are given, not by task performance.

Table S6: Comparing zero-shot model performances across different metrics on FS-Mol. In zero-shot mode no "test" molecules are provided. Results that are both the best and statistically significant (Welch's t-test[S34] $\alpha = 0.05$ with Benjamini-Hochberg[S35] multiple test correction) are highlighted in bold.

Model	Mode	ROC AUC (%)	PR AUC (%)	ΔPR AUC (%)
**TwinBooster**	zero-shot	$\mathbf{71.11 \pm 0.29}$	$\mathbf{68.56 \pm 0.24}$	$\mathbf{20.84 \pm 0.24}$
**CLAMP**‡ [S16]	zero-shot	69.26 ± 0.20	66.55 ± 0.20	19.37 ± 0.20

Table S7: All p-value results are from pairwise tests using the Wilcoxon test with Benjamini-Hochberg correction against the FS-Mol baselines. Only means of PR AUC and ΔPR AUC per task are used for the Wilcoxon test. Significance is indicated at $p_{\text{corr}} < \alpha$, where $\alpha = 0.05$. The Benjamini-Hochberg correction is used to correct for multiple testing.[S35]

Model	# support	p	p_{corr}	Significant	Outperformed
GNN-MT	16	3.20×10^{-13}	1.14×10^{-12}	True	True
	32	1.94×10^{-10}	3.74×10^{-10}	True	True
	64	3.64×10^{-6}	4.79×10^{-6}	True	True
	128	3.16×10^{-1}	3.16×10^{-1}	False	False
GNN-ST	16	3.59×10^{-19}	8.98×10^{-18}	True	True
	32	8.05×10^{-18}	7.50×10^{-17}	True	True
	64	9.00×10^{-18}	7.50×10^{-17}	True	True
	128	4.07×10^{-15}	1.70×10^{-14}	True	True
GNN-MAML	16	6.68×10^{-13}	2.09×10^{-12}	True	True
	32	2.70×10^{-11}	5.62×10^{-11}	True	True
	64	6.79×10^{-9}	1.13×10^{-8}	True	True
	128	3.70×10^{-4}	4.41×10^{-4}	True	True
MAT	16	2.33×10^{-17}	1.45×10^{-16}	True	True
	32	3.91×10^{-15}	1.70×10^{-14}	True	True
	64	9.91×10^{-12}	2.25×10^{-11}	True	True
	128	7.71×10^{-7}	1.13×10^{-6}	True	True
RF	16	1.72×10^{-12}	4.30×10^{-12}	True	True
	32	1.76×10^{-9}	3.15×10^{-9}	True	True
	64	6.78×10^{-6}	8.47×10^{-6}	True	True
	128	1.46×10^{-2}	1.66×10^{-2}	True	True
PN	16	4.78×10^{-2}	4.98×10^{-2}	True	True
	32	2.92×10^{-2}	3.17×10^{-2}	True	False
	64	8.34×10^{-7}	1.16×10^{-6}	True	False
	128	1.04×10^{-12}	2.90×10^{-12}	True	False
CLAMP	0	1.58×10^{-8}	2.46×10^{-8}	True	True

Comparing zero-shot performances of 10 replicates with or without conformal prediction

on FS-Mol yields $p \simeq 0.0020$, for ROC AUC, PR AUC and ΔPR AUC on a Wilcoxon test.[S34] Significance is indicated at $p < \alpha$, where $\alpha = 0.05$.

The performance across different protein super-families of the test set from FS-Mol is reported in table S8. We would also like to add that the predictions made by TwinBooster should not be interpreted as a dose/concentration, but as a zero-shot classification.

Table S8: Average performance of TwinBooster per protein super-family present in the test set of FS-Mol.

**Protein super-family**	**ROC AUC (%)**	**PR AUC (%)**	**ΔPR AUC (%)**	**# AIDs**
kinase	75.32	72.82	24.47	78
epigenetic	62.43	62.44	12.46	7
protease	61.86	59.16	13.74	7
undefined enzyme/protein	67.54	62.49	17.97	7
transferase	61.44	59.88	13.60	6
phosphatase	64.81	57.13	16.00	4
cytochrome	72.11	72.63	23.05	2
hydrolase	58.44	54.53	8.39	2
lyase	69.49	67.45	17.61	2
reductase	56.79	53.58	8.73	2
transporter	52.78	51.89	2.29	2
aminoacyltransferase	81.62	81.93	31.93	1
cytosolic	61.13	57.49	7.86	1
nuclear	67.93	69.27	19.57	1

Exploring the challenges of interfering compounds in HTS

We performed a correlation analysis between the predictions from TwinBooster and the frequent hitter probability according to HitDexter 3.[S6] The mean Pearson correlation coefficient[S36] for the target-based assay dataset model is $\rho \simeq -0.0353$, for the cell-based model is $\rho \simeq 0.0536$ and for the extended cell-based model is $\rho \simeq 0.0890$, indicating no correlation, respectively.

In addition, GSK and REOS structural alerts are used to identify possible molecular artefacts.[S5] A ROC AUC $\simeq 0.5179$, while a ROC AUC $= 0.5$ denotes randomness, shows that the predictions do not correlate with structural alerts, the same conclusion can be drawn from fig. S2, where no trend towards a class can be observed.

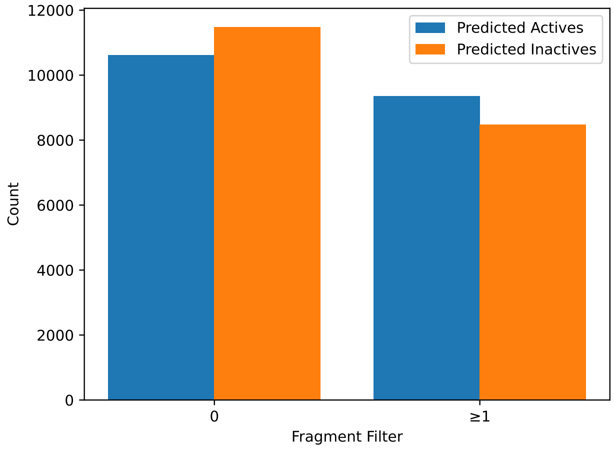

Figure S2: Number of molecules, according to their predicted class, which have no or one to many structural alerts. The class threshold is set to the median of the predictions.

As a further ablation, we built a model trained only on the kinase information contained in the FS-Mol training set. TwinBooster is able to significantly outperform the kinase-only RF model on the non-kinase test tasks of FS-Mol (refer to fig. S3). The kinase-only model is only able to perform slightly above random with a ΔPR AUC $= 5.33 \pm 8.17\,\%$, in contrast to TwinBooster with a ΔPR AUC $= 14.42 \pm 1.04\,\%$.

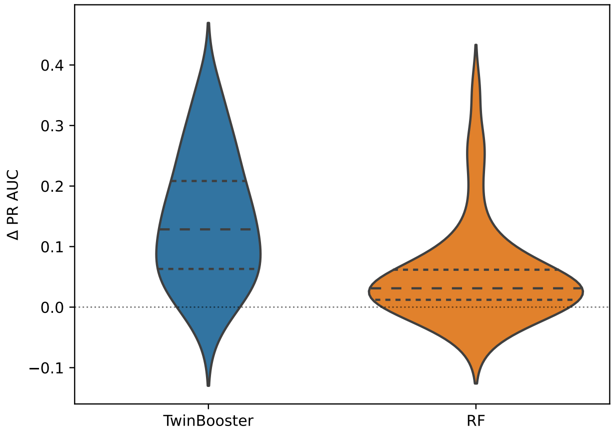

Figure S3: Performance of the TwinBooster compared to a kinase-only RF model on the non-kinase test tasks of FS-Mol. We performed the Wilcoxon test.[S34] Significance is indicated at $p < \alpha$, where $p \simeq 6.1 \times 10^{-5}$ and $\alpha = 0.05$.

Ablation study

The LSA model[S15] is pretrained on the PubChem corpus. LSA is chosen because of its non-LLM nature and its use in literature.[S16] This process is carried out in a manner similar to the approach used in Seidl *et al.*, using the Python packages `TfidfVectorizer` and `TruncatedSVD`.[S7,S16] The resulting text embeddings for each bioassay have a dimensionality of 355.

In parallel, the hyperparameter optimisation for the GBM is performed in an analogous way as described in the main text.

The p-values of the statistical tests using the Wilcoxon test (table S9) with with Benjamini-Hochberg multiple test correction are provided in table S10.[S35]

Table S9: Performance of different ablation experiments on FS-Mol. All results are tested pairwise using the Wilcoxon test with Benjamini-Hochberg multiple test correction.[S34,S35] 10 replicates each are performed. Significance in Wilcoxon[S34] test is indicated at $\alpha = 0.05$ for all results except those in italics, which are not significant. Results that are both the best and statistically significant are highlighted in bold.

	**TwinBooster**	**ECFP + PubChemDeBERTa**	**ECFP + LSA**
ROC AUC (%)	*71.11 ± 0.29*	*70.88 ± 0.27*	70.20 ± 0.22
PR AUC (%)	**68.57 ± 0.24**	68.13 ± 0.29	67.51 ± 0.21
ΔPR AUC (%)	**20.84 ± 0.24**	20.41 ± 0.29	19.78 ± 0.21

Table S10: All p-value results are from pairwise tests using the Wilcoxon test with with Benjamini-Hochberg multiple test correction of different ablation experiments on FS-Mol.[S35] 10 replicates each are performed. Significance is indicated at $p_{\text{corr}} < \alpha$, where $\alpha = 0.05$. If $p_{\text{corr}} \geq \alpha$, values are in italics.

p	**TwinBooster vs. ECFP + PubChemDeBERTa**	**TwinBooster vs. ECFP + LSA**	**ECFP + PubChemDeBERTa vs. ECFP + LSA**
ROC AUC	*0.1055*	0.0020	0.0020
PR AUC	0.0059	0.0020	0.0020
ΔPR AUC	0.0059	0.0020	0.0020
p_{corr}			
ROC AUC	*0.1055*	0.0029	0.0029
PR AUC	0.0066	0.0029	0.0029
ΔPR AUC	0.0066	0.0029	0.0029

As an additional experiment, we explored one-hot encoding of assay type and protein family. It should be noted, however, that one-hot encoding faces several challenges in the context of zero-shot molecular property prediction, as it limits the ability of the model to predict only targets seen during training, as these cannot be encoded in an informative manner. Another requirement is the need for labelled tabular data, which is generally scarce in drug discovery. It can only use the information from 33 % of all tasks from FS-Mol, as these are only annotated. This is why the kinase-only model should not be directly compared with the above results. The performance is ΔPR AUC $= 19.20 \pm 0.09\,\%$. Therefore, the one-hot-encoding-based model is outperformed by the zero-shot-capable baseline, where LSA text embeddings are added (Welch's t test,[S34] $p < \alpha$, where $p \simeq 2.3 \times 10^{-7}$ and $\alpha = 0.05$). TwinBooster and the LSA text embedding baselines outperform the one-hot encoded model, can use more training data because they do not require tabular annotation, and perform zero-shot predictions.

Conformal prediction

A comparison of zero-shot performances with or without conformal prediction on FS-Mol is shown in table S11.

Table S11: Comparing zero-shot performances with or without conformal prediction on FS-Mol. The confidence level is set to $\epsilon = 0.80$. 10 replicates each are performed. Results that are both the best and statistically significant (Wilcoxon test,[S34] $\alpha = 0.05$) are highlighted in bold.

TwinBooster	Conformal Prediction ✗	Conformal Prediction ✓
ROC AUC (%)	71.11 ± 0.29	$\mathbf{73.76 \pm 0.30}$
PR AUC (%)	68.56 ± 0.24	$\mathbf{71.04 \pm 0.31}$
ΔPR AUC (%)	20.84 ± 0.24	$\mathbf{22.81 \pm 0.30}$

Prompt engineering

To analyse the contributions of the different parts of the textual description, we analyse AID 507074, which is included in the FS-Mol test set. The PubChem entry from which our textual

data originates contains the title and description (sentences 1–8).[S3] The methods information is excluded, but can be found in the corresponding publication (sentences 9–12).[S37] Both text sources are combined and performance is assessed for each trimmed text description, starting with the first sentence and adding one sentence at a time. We colour the text according to ΔPR AUC values, so the change in contribution can be oberseverd while reading fig. S4.

(1) Inhibition of recombinant PI3Kdelta by radioactive phosphotransfer assay in presence of 10 uM ATP. (2) The clinical success of multitargeted kinase inhibitors has stimulated efforts to identify promiscuous drugs with optimal selectivity profiles. (3) It remains unclear to what extent such drugs can be rationally designed, particularly for combinations of targets that are structurally divergent. (4) Here we report the systematic discovery of molecules that potently inhibit both tyrosine kinases and phosphatidylinositol-3-OH kinases, two protein families that are among the most intensely pursued cancer drug targets. (5) Through iterative chemical synthesis, X-ray crystallography and kinome-level biochemical profiling, we identified compounds that inhibit a spectrum of new target combinations in these two families. (6) Crystal structures revealed that the dual selectivity of these molecules is controlled by a hydrophobic pocket conserved in both enzyme classes and accessible through a rotatable bond in the drug skeleton. (7) We show that one compound, PP121, blocks the proliferation of tumor cells by direct inhibition of oncogenic tyrosine kinases and phosphatidylinositol-3-OH kinases. (8) These molecules demonstrate the feasibility of accessing a chemical space that intersects two families of oncogenes. (9) For IC50 value determinations, purified kinase domains were incubated with inhibitors at two-fold or four-fold dilutions over a concentration range of 50 µM to 0.001 µM or with vehicle (0.1% DMSO) in the presence of 10 µM ATP, 2.5 µCi of [γ-32P]ATP and substrate. (10) Reactions were terminated by spotting onto nitrocellulose or phosphocellulose membranes, depending on the substrate; this membrane was then washed five or six times to remove unbound radioactivity and dried. (11) Transferred radioactivity was quantitated by phosphorimaging, and IC50 values were calculated by fitting the data to a sigmoidal dose-response using Prism (GraphPad). (12) Single concentration kinome profiling was performed using the Invitrogen SelectScreen assay.

Figure S4: Text contribution according to zero-shot performance of AID 507074. The lower the value, the lighter the colour (min(ΔPR AUC) = 0.0879) and the higher the value, the darker the colour (max(ΔPR AUC) = 0.3824). Sentences 1–8 are found in the PubChem entry and 9–12 are added from the publication.[S37]

In general, providing the model with more textual detail helps to improve performance. It can be observed that sentences containing information that is rarely present in the context of PubChem assays, such as information about crystal structures (e.g. sentence 6), lead to a decrease in performance. On the contrary, experimental details such as substrate, cell line and conditions seem to increase performance (sentences 7–9). After sentence 10, performance

seems to plateau. This could be explained by the token length of the fine-tuned LLM. When a string contains more than 128 words, the overhanging tokens are mean averaged in the embedding space, potentially reducing the information carried by the text embeddings. We reach the token length after sentence 6 and a second time after sentence 10, suggesting that text descriptions over 256 words may not be beneficial for predictive performance.

The order of the sentences does not contribute to the change in performance, which can be explained by the training procedure of the LLM, since the reinforcement is performed by shuffling the sentences. Due to the computational cost of predicting performance for all text permutations, this experiment was not conducted ($12! \simeq 480 \times 10^6$ combinations).

Case study

Figure S5 shows an enrichment of the number of unique Murcko scaffolds identified by TwinBooster and their proportional representation from the primary screen. This indicates that TwinBooster does not prioritise certain scaffolds, but enriches the scaffold diversity compared to random scaffold selection. Highlighting the importance of scaffold variability in early drug development, this increase in diversity is critical to the identification of potential leads.

The Mann-Whitney U test[S34] is conducted to asses the difference in Tanimoto similarity score and reveals $p \simeq 3.5e - 48$.

Table S12: Performance metrics of TwinBooster on the primary screen with or without conformal prediction. The confidence level is set to $\epsilon = 0.80$.

**TwinBooster**	**Conformal Prediction** ✗	✓
ROC AUC (%)	58.82	62.02
PR AUC (%)	7.10	11.64
ΔPR AUC (%)	3.34	5.73

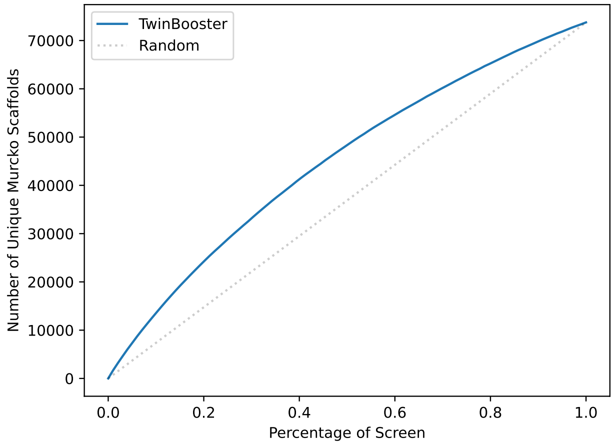

Figure S5: Number of detected unique Murcko scaffolds in relation to the percentage of scaffolds from the primary screen.

Primary screen This represents title, description and protocol is used for the zero-shot prediction of the primary screen (AID 2732[§]), based on the PubChem entry:[S3]

> HTS for small molecule inhibitors of CHOP to regulate the unfolded protein response to ER stress. Many genetic and environmental diseases result from defective protein folding within the secretory pathway so that aberrantly folded proteins are recognized by the cellular surveillance system and retained within the endoplasmic reticulum (ER). Under conditions of malfolded protein accumulation, the cell activates the Unfolded Protein Response (UPR) to clear the malfolded proteins, and if unsuccessful, initiates a cell death response. Preliminary studies have shown that CHOP is a crucial factor in the apoptotic arm of the UPR; XBP1 activates genes encoding ER protein chaperones and thereby mediates the adaptive UPR response to increase clearance of malfolded proteins. Inhibition of CHOP is hypothesized to enhance survival by preventing UPR programmed cell death. There are currently no known small molecule CHOP inhibitors either for laboratory or clinical use. To identify small molecule inhibitors of the UPR pathway, mediated by CHOP, a cell-based luciferase reporter assay using

[§] `https://pubchem.ncbi.nlm.nih.gov/bioassay/2732`

stably transfected CHO-K1 cells with luciferase driven by the CHOP promoter has been developed. The assay have been optimized and validated in 384-well format and used to screen for inhibitors of tunicamycin-induced CHOP in HTS. These identified compounds will have potential therapeutic application to diverse disease states ranging from diabetes, Alzheimer's disease, and Parkinson's disease, to hemophilia, lysosomal storage diseases, and alpha-1 antitrypsin deficiency. Reagents: 1. Cell line: CHO-CHOP cells with a luciferase reporter driven by the CHOP promoter (provided by assay PI) 2. Cell growth media (Ham's F12 + Glutamax, 10% FBS, 1X non-essential amino acids, and penicillin:streptomycin) (Invitrogen) 3. Tunicamycin (Calbiochem) 4. SteadyGlo reagent (Promega) Protocol: 1. 40 uL of medium containing CHO-CHOP cells (3000-4000) were dispensed to 384 well white opaque plates (Corning #3570) using a Multidrop combi (Thermo-Fisher Scientific). Plates were then incubated for 24 hrs at 37 degrees C, 5% CO2. 2. 0.5 uL of library compounds (1 mM in DMSO) was added to wells using Sciclone (Caliper LifeSciences). The final concentration of compound is 10 uM. 3. 10 uL of fresh medium containing tunicamycin (Tm) (2.0 ug/ml, final concentration,) was then added and the plates were incubated for 15-18 hrs. 4. Medium was aspirated with an Elx405 plate washer (BioTek), leaving 10 uL of medium in the well. 10 uL of Steady-Glo was added to each well using a multildrop combi. 5. Luminescence signal was measured on an Envision Multilable plate reader (PerkinElmer).

Investigating molecular structures We have examined various molecular structures in fig. S6 presented by our selected case study. As can be seen, the predicted top active molecules are indeed TPs and were also correctly prioritised in the primary screen. Structurally, the molecules differ significantly and are not derivatives of each other. This is also evident in fig. S5, where TwinBooster also shows that scaffold diversity is enriched compared to

random selection. Looking at the fig. S6b, there is again no obvious structural similarity. One molecule can be identified as a FNs, while all other structures are correctly classified.

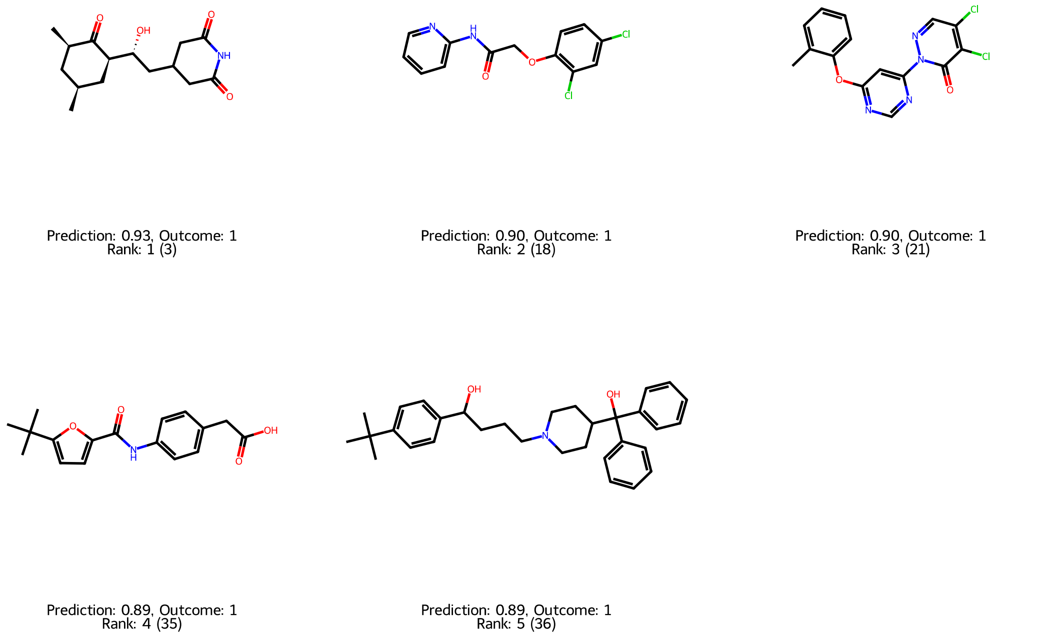

(a) Top 5

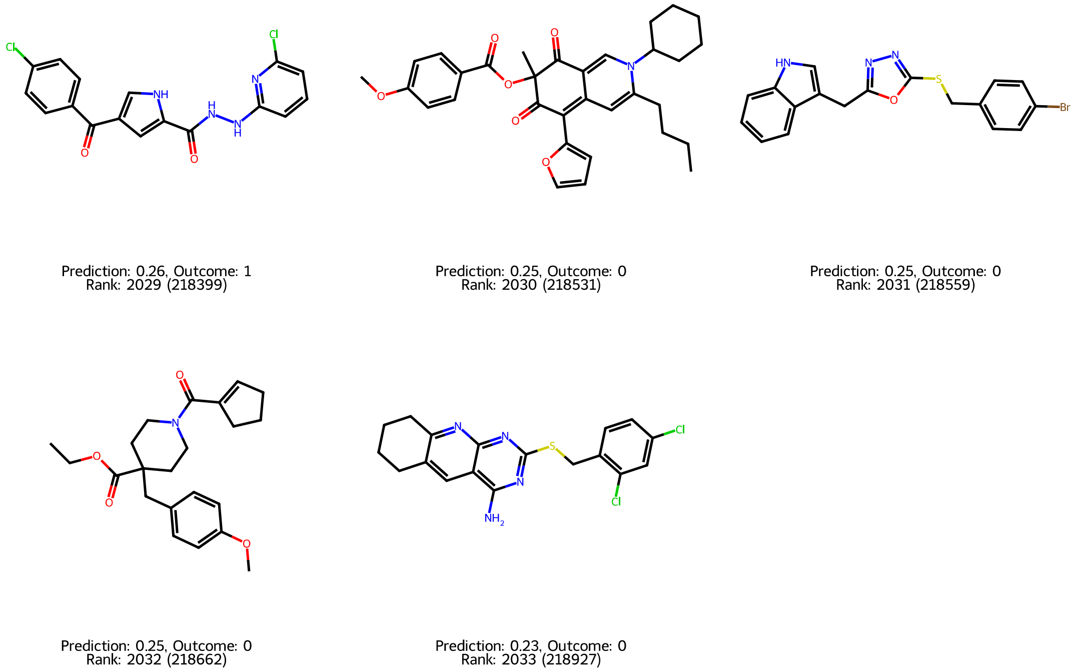

(b) Bottom 5

Figure S6: Molecular structures of the top and bottom five compounds ranked by TwinBooster. We show the predicted likelihood of being active in the CHOP pathway, the confirmatory assay outcome and the rank in the confirmatory screen (as well as in the primary screen).

References

(S1) Stanley, M.; Bronskill, J. F.; Maziarz, K.; Misztela, H.; Lanini, J.; Segler, M.; Schneider, N.; Brockschmidt, M. FS-Mol: A Few-Shot Learning Dataset of Molecules. Thirty-Fifth Conference on Neural Information Processing Systems Datasets and Benchmarks Track (Round 2). 2021.

(S2) Mendez, D.; Gaulton, A.; Bento, A. P.; Chambers, J.; De Veij, M.; Félix, E.; Magariños, M. P.; Mosquera, J. F.; Mutowo, P.; Nowotka, M. *et al.* ChEMBL: Towards Direct Deposition of Bioassay Data. *Nucleic acids research* **2019**, *47*, D930–D940.

(S3) Kim, S.; Chen, J.; Cheng, T.; Gindulyte, A.; He, J.; He, S.; Li, Q.; Shoemaker, B. A.; Thiessen, P. A.; Yu, B. *et al.* PubChem 2023 Update. *Nucleic Acids Res.* **2023**, *51*, D1373–D1380.

(S4) van Rossum, G. Python Tutorial. **1995**,

(S5) Landrum, G.; Tosco, P.; Kelley, B.; sriniker,; gedeck,; NadineSchneider,; Vianello, R.; Ric,; Dalke, A.; Cole, B. *et al.* Rdkit/Rdkit: 2020_03_1 (Q1 2020) Release. Zenodo, 2020.

(S6) Stork, C.; Chen, Y.; Šícho, M.; Kirchmair, J. Hit Dexter 2.0: Machine-Learning Models for the Prediction of Frequent Hitters. *J. Chem. Inf. Model.* **2019**, *59*, 1030–1043.

(S7) Pedregosa, F.; Varoquaux, G.; Gramfort, A.; Michel, V.; Thirion, B.; Grisel, O.; Blondel, M.; Prettenhofer, P.; Weiss, R.; Dubourg, V. *et al.* Scikit-learn: Machine Learning in Python. *Journal of Machine Learning Research* **2011**, *12*, 2825–2830.

(S8) Tharwat, A. Classification Assessment Methods. *Appl. Comput. Inf.* **2020**, *17*, 168–192.

(S9) He, P.; Gao, J.; Chen, W. DeBERTaV3: Improving DeBERTa Using ELECTRA-Style Pre-Training with Gradient-Disentangled Embedding Sharing. 2023.

(S10) He, P.; Liu, X.; Gao, J.; Chen, W. DeBERTa: Decoding-enhanced BERT with Disentangled Attention. 2021.

(S11) Wolf, T.; Debut, L.; Sanh, V.; Chaumond, J.; Delangue, C.; Moi, A.; Cistac, P.; Rault, T.; Louf, R.; Funtowicz, M. *et al.* Transformers: State-of-the-Art Natural Language Processing. Proceedings of the 2020 Conference on Empirical Methods in Natural Language Processing: System Demonstrations. Online, 2020; pp 38–45.

(S12) Akiba, T.; Sano, S.; Yanase, T.; Ohta, T.; Koyama, M. Optuna: A Next-generation Hyperparameter Optimization Framework. 2019.

(S13) Devlin, J.; Chang, M.-W.; Lee, K.; Toutanova, K. BERT: Pre-training of Deep Bidirectional Transformers for Language Understanding. 2019.

(S14) Lee, J.; Yoon, W.; Kim, S.; Kim, D.; Kim, S.; So, C. H.; Kang, J. BioBERT: A Pre-Trained Biomedical Language Representation Model for Biomedical Text Mining. *Bioinformatics* **2020**, *36*, 1234–1240.

(S15) Deerwester, S.; Dumais, S. T.; Furnas, G. W.; Landauer, T. K.; Harshman, R. Indexing by Latent Semantic Analysis. *Journal of the American Society for Information Science* **1990**, *41*, 391–407.

(S16) Seidl, P.; Vall, A.; Hochreiter, S.; Klambauer, G. Enhancing Activity Prediction Models in Drug Discovery with the Ability to Understand Human Language. 2023.

(S17) Jelinek, F.; Mercer, R. L.; Bahl, L. R.; Baker, J. K. Perplexity—a Measure of the Difficulty of Speech Recognition Tasks. *The Journal of the Acoustical Society of America* **1977**, *62*, S63.

(S18) Meister, C.; Cotterell, R. Language Model Evaluation Beyond Perplexity. Proceedings of the 59th Annual Meeting of the Association for Computational Linguistics and the

11th International Joint Conference on Natural Language Processing (Volume 1: Long Papers). Online, 2021; pp 5328–5339.

(S19) Zbontar, J.; Jing, L.; Misra, I.; LeCun, Y.; Deny, S. Barlow Twins: Self-Supervised Learning via Redundancy Reduction. 2021.

(S20) Ioffe, S.; Szegedy, C. Batch Normalization: Accelerating Deep Network Training by Reducing Internal Covariate Shift. 2015.

(S21) Agarap, A. F. Deep Learning Using Rectified Linear Units (ReLU). 2019.

(S22) Ramachandran, P.; Zoph, B.; Le, Q. V. Searching for Activation Functions. 2017.

(S23) Paszke, A.; Gross, S.; Massa, F.; Lerer, A.; Bradbury, J.; Chanan, G.; Killeen, T.; Lin, Z.; Gimelshein, N.; Antiga, L. *et al.* PyTorch: An Imperative Style, High-Performance Deep Learning Library. 2019.

(S24) Loshchilov, I.; Hutter, F. Decoupled Weight Decay Regularization. 2019.

(S25) Tishby, N.; Zaslavsky, N. Deep Learning and the Information Bottleneck Principle. 2015.

(S26) Ke, G.; Meng, Q.; Finley, T.; Wang, T.; Chen, W.; Ma, W.; Ye, Q.; Liu, T.-Y. LightGBM: A Highly Efficient Gradient Boosting Decision Tree. Advances in Neural Information Processing Systems. 2017.

(S27) Lindauer, M.; Eggensperger, K.; Feurer, M.; Biedenkapp, A.; Deng, D.; Benjamins, C.; Ruhopf, T.; Sass, R.; Hutter, F. SMAC3: A Versatile Bayesian Optimization Package for Hyperparameter Optimization. 2022.

(S28) Fawcett, T. An Introduction to ROC Analysis. *Pattern Recognit. Lett.* **2006**, *27*, 861–874.

(S29) *Learning from Imbalanced Data Sets*; Springer Science+Business Media: New York, NY, 2018.

(S30) Branco, P.; Torgo, L.; Ribeiro, R. A Survey of Predictive Modelling under Imbalanced Distributions. 2015.

(S31) Piryonesi, S. M.; El-Diraby, T. E. Data Analytics in Asset Management: Cost-Effective Prediction of the Pavement Condition Index. *J. Infrastruct. Syst.* **2020**, *26*, 04019036.

(S32) Cortés-Ciriano, I.; Bender, A. Concepts and Applications of Conformal Prediction in Computational Drug Discovery. 2019.

(S33) Stone, M. Cross-Validatory Choice and Assessment of Statistical Predictions. *Journal of the Royal Statistical Society. Series B (Methodological)* **1974**, *36*, 111–147.

(S34) Virtanen, P.; Gommers, R.; Oliphant, T. E.; Haberland, M.; Reddy, T.; Cournapeau, D.; Burovski, E.; Peterson, P.; Weckesser, W.; Bright, J. *et al.* SciPy 1.0: Fundamental Algorithms for Scientific Computing in Python. *Nat. Methods* **2020**, *17*, 261–272.

(S35) Seabold, S.; Perktold, J. statsmodels: Econometric and statistical modeling with python. 9th Python in Science Conference. 2010.

(S36) Pearson, K. Note on Regression and Inheritance in the Case of Two Parents. *Proceedings of the Royal Society of London Series I* **1895**, *58*, 240–242.

(S37) Apsel, B.; Blair, J. A.; Gonzalez, B.; Nazif, T. M.; Feldman, M. E.; Aizenstein, B.; Hoffman, R.; Williams, R. L.; Shokat, K. M.; Knight, Z. A. Targeted Polypharmacology: Discovery of Dual Inhibitors of Tyrosine and Phosphoinositide Kinases. *Nat. Chem. Biol.* **2008**, *4*, 691–699.
